# Factors affecting subjective cognitive decline: an automated machine learning approach

**DOI:** 10.3389/fnins.2025.1658247

**Published:** 2025-11-18

**Authors:** Yunting Xu, Jiaxing Zheng, Yuting Tang, Kaiwen Chen, Liyan Wu, Wangxiang Mai, Zhuoming Chen

**Affiliations:** 1 Department of Rehabilitation Medicine, The First Affiliated Hospital of Jinan University, Guangzhou, Guangdong, China; 2 School of Mathematics, South China University of Technology, Guangzhou, Guangdong, China

**Keywords:** subjective cognitive decline, machine learning, information overload, self-perception, energy levels

## Abstract

**Background:**

This study aims to develop a screening model for subjective cognitive decline (SCD) based on machine learning techniques.

**Methods:**

A retrospective cohort study collected clinical psychological factor data from the “Active Health” screening app under the National Key R&D Program. The final dataset included 598 samples, with an SCD incidence rate of 26.12%. The data were randomly divided into a training set (*n* = 418). A validation set (*n* = 180) at a ratio of 7:3. In the training set, prediction models for SCD were constructed using logistic regression (LR), Naive Bayes, support vector machine (SVM), decision tree, and neural network algorithms. Model performance on the validation set was assessed by calculating the area under the ROC curve (AUC), accuracy, sensitivity, specificity, precision, recall, and F1 score. SHAP values were used for model interpretability analysis.

**Results:**

The SVM model showed good performance in the training set, with an AUC of 0.82, indicating strong predictive ability. Information Overload (IO), Self-Perception (SP), Energy Level (EL), Depressive Emotion (DE), Gender (SEX), Risk Decision (RD), and Short-Term Memory (STM) were important feature variables for SCD occurrence.

**Conclusion:**

This study successfully developed an SVM-based model for screening the risk of SCD. The SVM model demonstrated superior predictive performance compared to Naïve Bayes, Decision Tree, Neural Network, and traditional LR models.

## 1 Introduction

Subjective cognitive decline (SCD) refers to an individual’s self-perceived decline in memory or other cognitive functions compared to their previous normal state, while objective neuropsychological assessments remain within normal limits (Ong et al., 2018). Studies have shown that the incidence of SCD among older adults aged 65 and above is approximately 28% (Jessen et al., 2023). For the elderly, SCD not only indicates a potential risk of cognitive impairment but also negatively impacts various aspects of daily life and psychological well-being, such as anxiety, depression, sleep disorders, and reduced willingness to engage in social activities (Janssen et al., 2022; Jessen et al., 2020). SCD is not a disease in itself, but rather a symptom that may reflect normal aging or serve as an early indicator of cognitive impairment or Alzheimer’s disease (AD) (Jessen et al., 2014). In the pathological progression of AD, SCD is often considered one of the clinical manifestations in the second stage (Pike et al., 2022). Multiple studies have found that compared to individuals without SCD, those with SCD have a significantly higher risk of developing dementia in the future, with a conversion rate to dementia of about 10% (Ross et al., 1982; Wang et al., 2020). Pathologically, SCD has been associated with varying degrees of amyloid-beta positivity, suggesting it may reflect early AD pathological changes (Itzhak et al., 1984; Janssen et al., 2022). Therefore, early identification of individuals with SCD is of great significance for studying the early pathological mechanisms of preclinical AD and reducing the incidence of AD (Wang et al., 2023).

In clinical practice, common cognitive assessments for SCD include screening tools such as the Mini-Mental State Examination (MMSE), Montreal Cognitive Assessment (MoCA), and Mini-Cog. Over the past decade, neuroimaging techniques have also been widely used to identify biomarkers related to early diagnosis of AD, including computed tomography (CT), magnetic resonance imaging (MRI), and positron emission tomography (PET) (Heser et al., 2019). However, these methods are costly, not widely accessible, and difficult to use as routine tools for large-scale population screening. Given that there is currently no effective cure for SCD (Jiang et al., 2022), early detection, diagnosis, and timely intervention are critically important. Numerous studies have demonstrated that the occurrence of SCD is associated with factors such as gender, sleep and mood disorders, memory, decision-making ability, depression, medication use, personality traits, and poor overall health (Bazgir et al., 2023; Choi et al., 2020; Grund and Rossi, 1981; Lee et al., 2020; Nelson et al., 2022; Pini and Wennberg, 2021). These factors show good predictive accuracy for subjective cognitive decline (Schweppe et al., 2022), but most current studies focus on single factors or traditional statistical models. Currently, most applications of machine learning in the early screening of SCD rely on digital biomarkers such as speech, gait, and eye movement signals to enable early risk identification (Ding et al., 2024; Hao et al., 2024). In addition, some researchers have combined magnetic resonance imaging (MRI) with clinical rating scales to predict Aβ positivity or subclinical abnormalities at the SCD stage (Jung et al., 2023). These advances highlight the great potential of machine learning in developing clinical tools to support disease screening and prediction.

This study aims to explore the effects of various factors, such as information overload, sleep quality, energy levels, concentration, short-term memory, self-perception, and long-term memory, on cognitive function by employing automated machine learning methods. By identifying and analyzing the interactions among these factors and their impact on cognition, the study provides novel perspectives and methodological approaches for advancing the understanding and enhancement of cognitive function. The findings hold substantial theoretical and practical significance.

## 2 Materials and methods

### 2.1 Materials

This study collected clinical data from 598 participants across 16 regions between August 3, 2022, and May 19, 2024, using the “Active Health” screening mini-program of the National Key R&D Program. Among them, 212 were male and 386 were female. The inclusion criteria were as follows: (1) age between 35 and 70 years; (2) normal consciousness and no communication barriers with the researchers; and (3) voluntary participation with electronic informed consent obtained. The exclusion criteria were: (1) diagnosis of cognitive-related diseases such as stroke, traumatic brain injury, or Parkinson’s disease; (2) experience of severe psychological stress or acute illness within the past 3 months; and (3) diagnosis of psychiatric or psychological disorders such as severe or mild dementia or depression.

The “Active Health” screening mini-program is part of the National Key Research and Development Program of China. It was developed under the Ministry of Science and Technology’s National Key Research and Development Project titled “Intelligent Adaptation and Demonstration Application of Assistive Devices for Daily Living and Motor Rehabilitation” (Project No. 2020YFC2005700). This program provides personalized rehabilitation interventions and assistive device adaptations for individuals with impairments or declines in activities of daily living, mobility, balance, motor function, speech, and cognition. It is especially designed for people with hypertension, diabetes, stroke, Parkinson’s disease, Alzheimer’s disease, cancer, and physical frailty. The program offers a range of functional services, including “Science Popularization,” “Functional Screening,” “Health Management,” and “Rehabilitation Assistive Devices.” Collected data include gender, age, energy level, sleep quality, attention, short-term memory, self-perception, long-term memory, hidden object recognition, spatial orientation, self-assessed risk decision-making, psychological evaluation, visual perception, and information overload and decision-making ability. All data were collected through self-administered online questionnaires using the “Active Health” mini-program, primarily completed on mobile devices, with optional access via tablets or computers. The questionnaire employed a step-by-step guided format with mandatory responses, and key items included consistency checks and explanatory prompts to minimize input errors and missing data. Participants could review and revise their answers before submission, after which the data were encrypted and uploaded to the server. Based on the Subjective Cognitive Decline Questionnaire (SCD-Q9) developed by Gifford et al. (2015), participants were classified into the SCD group or the healthy control (HC) group according to whether their scores exceeded 5 points (Gao et al., 2024). All participants provided informed consent in accordance with the Declaration of Helsinki, and the study was approved by the Ethics Committee of the First Affiliated Hospital of Jinan University (Approval No. KY-2024-013).

### 2.2 Data preprocessing

Basic patient information and factor scores were exported from the backend of the “Active Health” screening mini-program under the National Key R&D Program^1^ for classification.

### 2.3 Factors associated with subjective cognitive decline

As shown in Table 1, Sleep Quality (SQ) refers to an individual’s self-perceived satisfaction with various aspects of their sleep experience. It encompasses four attributes: sleep efficiency, sleep latency, sleep duration, and awakenings after sleep onset (Nelson et al., 2022). Energy Level (EL) refers to an individual’s state of energy and vitality during a specific period. It reflects a person’s physiological and psychological energy reserves, as well as their ability to cope with daily activities, tasks, and stress (Grund and Rossi, 1981). Attention refers to the ability of an individual to allocate mental and physical resources toward a specific task, activity, or object over a given period, and it constitutes a fundamental component of cognitive function (Bazgir et al., 2023). Short-Term Memory (STM) is defined as the capacity to temporarily store and manipulate information over brief intervals, typically ranging from a few seconds to several tens of seconds (Schweppe et al., 2022). Self-perception refers to an individual’s awareness and understanding of their traits, behaviors, emotions, abilities, attitudes, and identity (Zhu et al., 2023). Long-Term Memory (LTM) denotes the ability to store and retrieve information over extended periods–from days to years or even a lifetime–and is characterized by its durability and stability (Grünbaum et al., 2021). Hidden Recognition refers to the cognitive process of uncovering implicit or underlying information, patterns, motives, or emotions. Location Recognition (LR) refers to the cognitive ability to accurately identify and determine the spatial, temporal, or contextual position of information, objects, or events (Lian et al., 2018). Risk Decision (RD) refers to the process of evaluating and selecting a course of action under conditions of uncertainty. This process involves weighing potential risks and benefits to achieve the optimal outcome or to minimize negative consequences (Pauley et al., 2011). Psychological Self-Assessment (PSA) refers to the process by which individuals evaluate and reflect on their psychological state, emotions, behaviors, attitudes, and mental traits through self-reporting methods (Kim et al., 2019). Visual Perception (VP) is the process by which individuals acquire, process, and interpret visual information from the environment through the visual system. It is a key component of the sensory and perceptual systems, involving the reception of light by the eyes and the brain’s interpretation of this information (Upadhyayula et al., 2023). Information Overload (IO) refers to a state in which the volume of information received exceeds an individual’s capacity to process it effectively, resulting in difficulties in understanding, analyzing, and making decisions (Liu et al., 2021). Depressive Emotion (DE) is a persistent and profound state of low mood, often accompanied by a loss of interest or pleasure in daily activities (Peters et al., 2021). Decision Bias (DB) refers to the systematic deviation of an individual’s judgments and decisions from rational and objective standards, influenced by cognitive, emotional, social, or other factors during the decision-making process (Larson and Hawkins, 2023). Decision Preference (DP) refers to an individual’s tendency or inclination toward a particular option when faced with multiple choices, based on personal values, beliefs, experiences, and emotions (Driever et al., 2022).

**TABLE 1:** Determinants of subjective cognitive function.

Parameter	Explanation
SQ	Sleep quality
EL	Energy level
Focus	
STM	Short-term memory
SP	Self-perception
LTM	Long-term memory
RH	Recognition hiding
LR	Location recognition
RD	Risk decision
PSA	Psychological self-assessment
VP	Visual perception
IO	Information overload
DE	Depressive emotion
DB	Decision bias
DP	Decision preference

### 2.4 Statistical analysis

Normality tests were first conducted for all continuous variables. Data conforming to a normal distribution were expressed as mean ± standard deviation (x̄ ± s), and group comparisons were performed using the *t*-test. For data that did not meet the assumptions of normality or homogeneity of variance, the Kruskal–Wallis H test was employed. Categorical variables were analyzed using the chi-square (χ^2^) test. All statistical analyses were conducted using R software (version 4.3.1), with a *p*-value of <0.05 considered statistically significant.

### 2.5 Model development

All continuous variables in this study were standardized using Z-score normalization, transforming each variable to have a mean of 0 and a standard deviation of 1. This normalization process aimed to eliminate scale inconsistencies across features and to center the data, thereby improving the efficiency and accuracy of model training.

To enhance the model’s generalizability and reduce the risk of overfitting, Least Absolute Shrinkage and Selection Operator (LASSO) regression was employed, based on methods used in prior studies (Lian et al., 2018), to identify and select significant predictors for model construction. The dataset comprising 598 participants was randomly divided into a training set (*n* = 418) and a testing set (*n* = 180) using a 70:30 split.

Five machine learning algorithms were employed to develop predictive models for SCD: logistic regression (LR), Naive Bayes, support vector machine (SVM), decision tree, and neural network. The neural network used in this study consisted of an input layer with seven neurons, a single hidden layer with three neurons, and an output layer designed to generate binary classification probabilities. The hidden layer employed a sigmoid activation function, and the output layer also used a sigmoid function to map results to probabilities between 0 and 1. Model training was conducted using the backpropagation algorithm for weight optimization, minimizing the sum of squared errors as the objective function to achieve parameter learning and obtain probabilistic outputs for binary classification. In the training set, dimensionality reduction and feature selection were performed using LASSO with cross-validation. Model complexity was determined according to the one-standard-error rule based on the cross-validation error curve, resulting in a more concise and generalizable subset of features. Continuous variables were standardized using Z-scores during the training phase, and the same selected features and data processing procedures were consistently applied across all classification models. Model performance was evaluated using AUC, accuracy, sensitivity, specificity, precision, recall, and F1 score. Clinical applicability was assessed through decision curve analysis, model calibration was examined using calibration curves, and interpretability was evaluated with Shapley Additive Explanations (SHAP), which quantified and visualized both the direction and magnitude of each feature’s contribution to the prediction outcomes.

In addition, permutation importance was used to assess feature contributions, with RMSE employed to measure probabilistic prediction error. Keeping the trained classification model fixed, each feature was randomly permuted one at a time, and the RMSE between the model’s predicted probabilities and the observed binary outcomes (coded as 0/1) was calculated on the validation set. In binary classification settings, the mean squared error (MSE) of probability predictions is equivalent to the Brier score, and RMSE, as its square root, serves as an appropriate measure of probabilistic prediction error. To reduce randomness, this procedure was repeated across validation folds during cross-validation, and the results were averaged. The baseline RMSE without permutation was also reported for comparison.

## 3 Results

### 3.1 Baseline characteristics

A total of 598 participants were included in the study, with 216 individuals in the SCD group and 382 in the healthy control (HC) group. Baseline characteristics compared between the two groups included gender, age, Education Level (EL), Sleep Quality (SQ), Focus, Short-Term Memory (STM), Self-Perception (SP), Long-Term Memory (LTM), Recognition Hiding (RH), Location Recognition (LR), Risk Decision (RD), Psychological Self- Assessment (PSA), Visual Perception (VP), and Information Overload (IO). The overall baseline characteristics of the two patient groups are shown in Table 2.

**TABLE 2:** Baseline characteristics of all participants.

Variables	Total (*n* = 598)	HC group (*n* = 382)	SCD group (*n* = 216)	Statistic	*P*
RH	35.77 ± 17.10	35.50 ± 16.79	36.25 ± 17.66	*t* = −0.52	0.606
DP	59.62 ± 23.28	59.82 ± 23.02	59.26 ± 23.77	*t* = 0.28	0.779
Age	46.12 ± 8.47	45.98 ± 8.45	46.36 ± 8.52	*t* = −0.52	0.606
SQ	62.87 ± 26.27	69.37 ± 23.11	51.39 ± 27.62	*t* = 8.10	<0.001
EL	58.72 ± 25.91	60.96 ± 27.21	54.76 ± 22.97	*t* = 2.96	0.003
Focus	60.81 ± 17.68	65.74 ± 16.60	52.10 ± 16.12	*t* = 9.75	<0.001
STM	64.98 ± 24.12	73.75 ± 17.95	49.47 ± 25.81	*t* = 12.25	<0.001
SP	53.65 ± 25.73	65.56 ± 19.17	32.61 ± 22.13	*t* = 19.08	<0.001
LTM	55.57 ± 16.60	62.39 ± 13.13	43.50 ± 15.20	*t* = 15.32	<0.001
LR	37.38 ± 12.27	37.24 ± 12.22	37.62 ± 12.36	*t* = −0.36	0.723
RD	43.38 ± 23.12	41.10 ± 23.16	47.42 ± 22.53	*t* = −3.24	0.001
PSA	57.23 ± 14.27	63.17 ± 11.73	46.73 ± 12.17	*t* = 16.24	<0.001
VP	29.65 ± 7.42	30.15 ± 7.42	28.75 ± 7.34	*t* = 2.23	0.026
IO	43.46 ± 20.76	32.47 ± 14.33	62.87 ± 15.48	*t* = −24.20	<0.001
DE	35.41 ± 20.22	27.41 ± 14.93	49.56 ± 20.62	*t* = −13.86	<0.001
DB	33.73 ± 19.32	34.35 ± 19.67	32.63 ± 18.67	*t* = 1.05	0.296

t, *t*-test; SD, standard deviation.

### 3.2 Feature selection results

**FIGURE 1:**
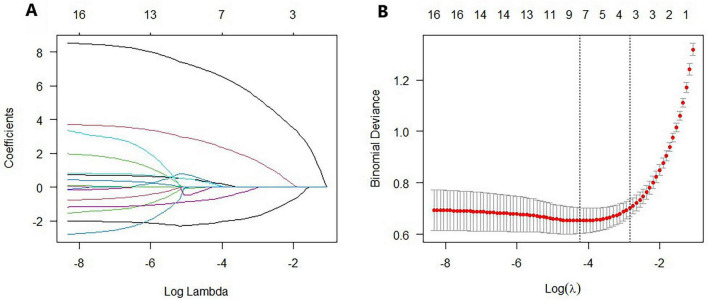
LASSO regression analysis results. **(A)** Coefficient profile plot of the LASSO model; **(B)** Cross-validation curve for tuning the lambda parameter.

Least Absolute Shrinkage and Selection Operator regression was performed on the training set to identify relevant feature variables. Variables with non-zero coefficients at the lambda value corresponding to the minimum standard error (Lambda. min) were selected for inclusion. The optimal lambda value, determined based on the minimum mean squared error, was 0.0144. At this threshold, the variables retained included Sex, Age, SQ, EL, Focus, STM, SP, LTM, RH, LR, RD, PSA, VP, IO, DE, DB, and DP. Among these, the final selected features were Sex, EL, STM, SP, RD, DE, and IO (Figure 1).

### 3.3 Model construction

**FIGURE 2:**
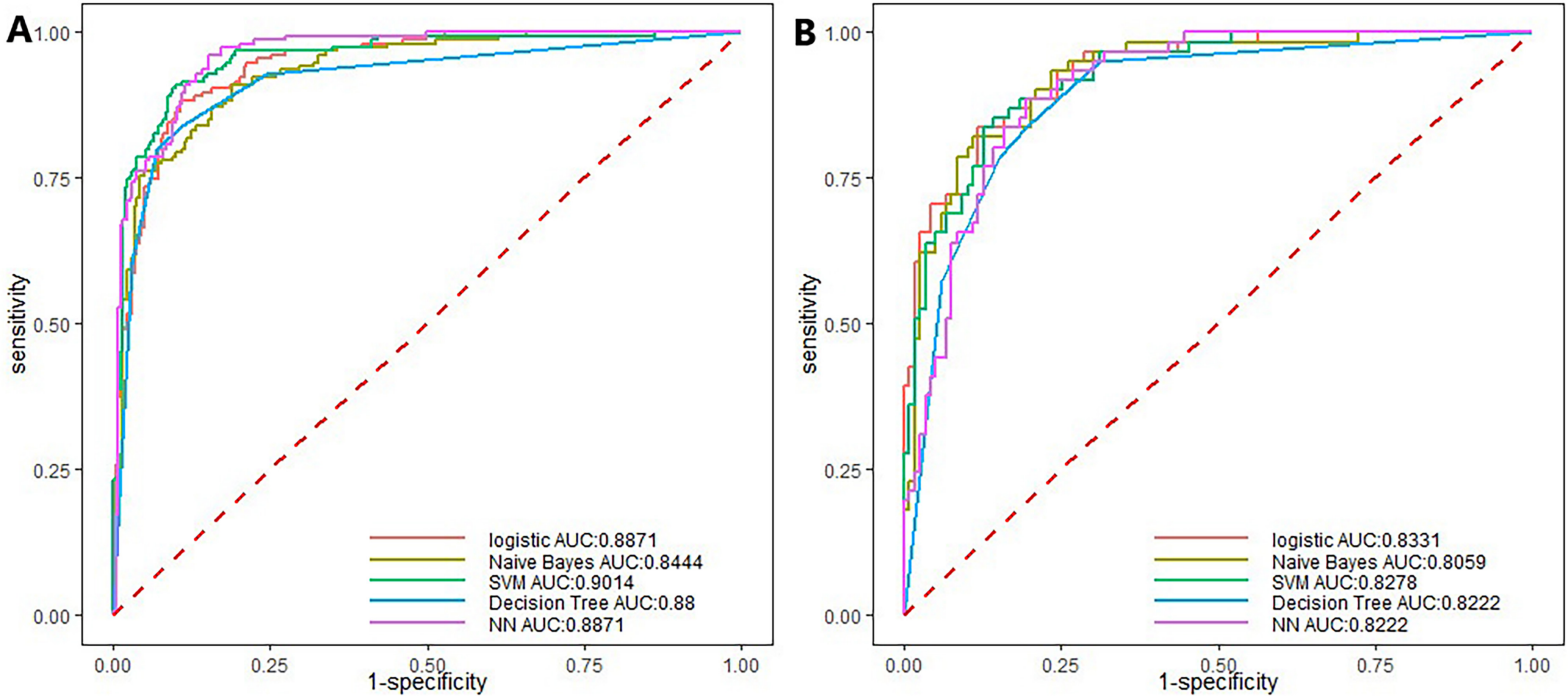
Receiver operating characteristic (ROC) curves of the machine learning models. **(A)** ROC curves for the training set; **(B)** ROC curves for the test set.

**FIGURE 3:**
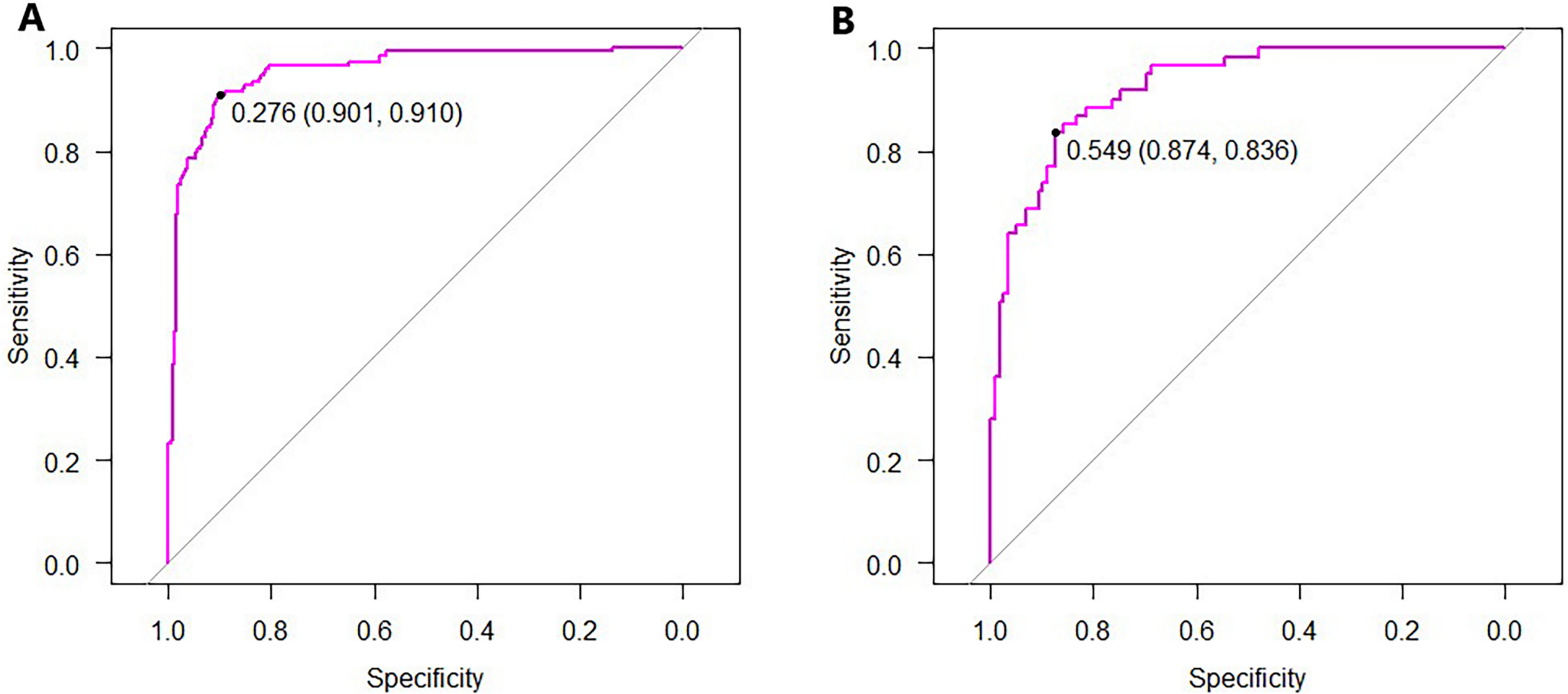
Receiver operating characteristic (ROC) curves of the SVM model. **(A)** ROC curve for the training set; **(B)** ROC curve for the test set.

In the training set, five machine learning algorithms–logistic regression (LR), Naive Bayes, support vector machine (SVM), decision tree, and neural network–were employed to develop predictive models based on the feature variables selected by LASSO regression at the lambda value corresponding to one standard error (Lambda.1se), including Sex, EL, STM, SP, RD, IO, and DE. Among these models, the SVM achieved the highest area under the curve (AUC) in both the training and internal validation sets, indicating superior performance and identifying it as the optimal model, as shown in Figures 2, 3A and Table 3.

**TABLE 3:** Comparison of predictive performance across multiple models.

Classification model	Accuracy	Sensitivity	Specificity	Pos pred value	Neg pred value	F1
**Training set**						
LR	0.89	0.88	0.89	0.83	0.93	0.86
Naive Bayes	0.85	0.91	0.81	0.74	0.94	0.82
SVM	0.90	0.91	0.90	0.84	0.94	0.88
Decision tree	0.88	0.80	0.93	0.87	0.89	0.84
Neural network	0.89	0.96	0.85	0.79	0.97	0.87
**Validation set**						
LR	0.84	0.87	0.82	0.72	0.92	0.79
Naive Bayes	0.81	0.95	0.74	0.65	0.97	0.77
SVM	0.83	0.89	0.81	0.70	0.93	0.78
Decision tree	0.83	0.79	0.83	0.73	0.89	0.76
Neural network	0.83	0.89	0.80	0.69	0.93	0.78

### 3.4 Model performance evaluation

**FIGURE 4:**
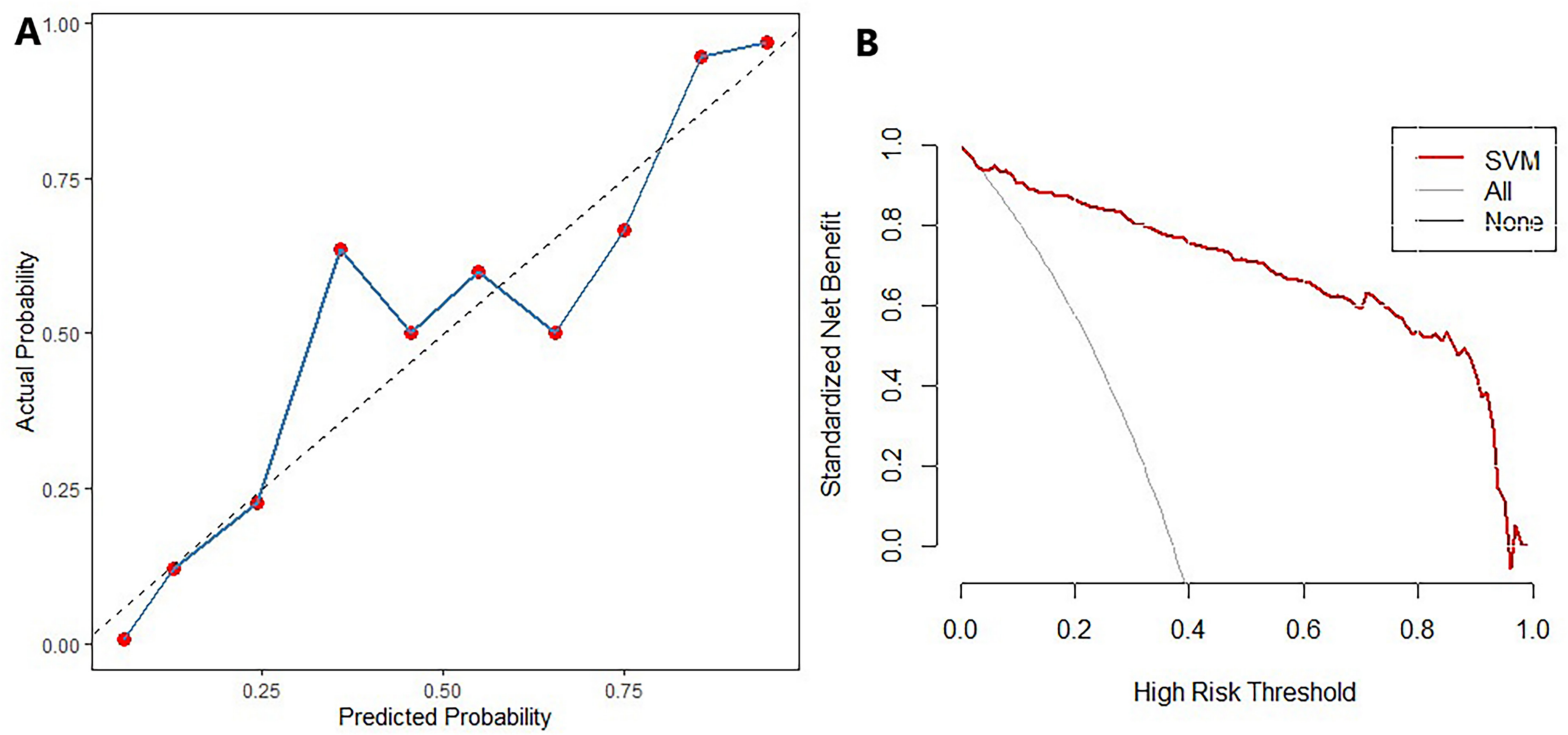
Calibration and decision curve analysis (DCA) of the SVM model in the test set. **(A)** Calibration curve; **(B)** Decision curve analysis curve.

The predictive performance of the support vector machine (SVM) algorithm for identifying individuals with SCD was evaluated on the test set. The SVM model achieved an area under the curve (AUC) of 0.82, an accuracy of 0.83, a sensitivity of 0.89, a specificity of 0.81, a positive predictive value (PPV) of 0.70, a negative predictive value (NPV) of 0.93, and an F1 score of 0.78. The ROC curve for the SVM model in the test set is presented in Figure 3B. Decision curve analysis (DCA) (Larson and Hawkins, 2023) was conducted to assess the clinical utility of the SVM model. As shown in Figure 4B, the SVM model demonstrated a broad range of net benefit, indicating strong clinical applicability. The calibration curve for the test set (Figure 4A) revealed good agreement between the predicted probabilities and the observed frequency of SCD, indicating that the SVM model was well-calibrated.

### 3.5 Model interpretability analysis

**FIGURE 5:**
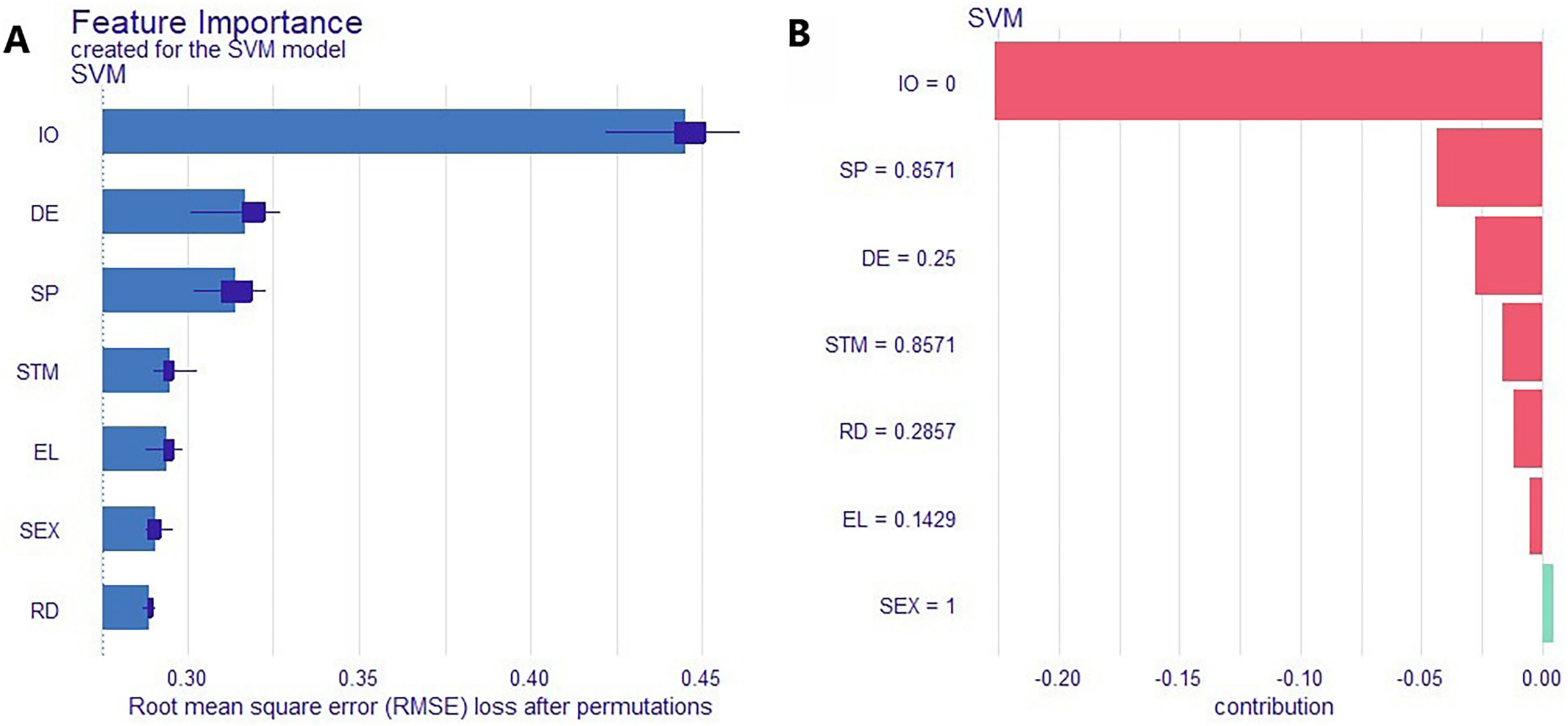
Shapley Additive Explanations (SHAP)-based interpretability analysis of the SVM model. **(A)** Ranked feature importance; **(B)** SHAP force plot for the first patient.

To better understand the key factors contributing to SCD and enhance the interpretability of the classification model, Shapley Additive Explanations (SHAP) were applied to the SVM model. As shown in Figure 5A, the SHAP summary plot ranks feature importance, with the top seven predictors being information overload (IO), self-perception (SP), Energy level (EL), Depressive Emotion (DE), sex (SEX), Risk Decision (RD), and Short-Term Memory (STM). These features can be considered the most influential contributors to SCD risk. Figure 5B presents the SHAP force plot for the first individual in the dataset, illustrating how each feature influenced the model’s prediction. IO had the greatest positive impact, with higher values increasing the probability of being classified as having SCD, indicating a strong positive association. Similar trends were observed for SP, DE, STM, EL, and RD. Regarding sex, the model indicated a higher predicted risk of SCD in females compared to males.

## 4 Discussion

This study examined the influence of clinical psychological factors on SCD and identified seven key predictors–Sex, Education Level (EL), Short-Term Memory (STM), Self-Perception (SP), Risk Decision (RD), Depressive Emotion (DE), and Information Overload (IO)–as significant contributors in the predictive model. These findings underscore the important role of psychological factors in assessing the risk of SCD. Among all variables, information overload (IO) was the most influential feature for prediction. Compared with cognitively normal individuals, those with subjective cognitive decline (SCD) experienced higher levels of IO, and the model consequently assigned a greater predicted probability of SCD. This finding aligns with previous theoretical and empirical research. When external information demands exceed an individual’s available attention and processing resources, IO is likely to occur, often resulting in confusion, fatigue, and avoidance, which in turn weaken the ability to filter and update key information (Eppler and Mengis, 2004; Roetzel, 2019). Eppler and Mengis (2004) also found that when the flow of information surpasses a person’s processing capacity, individuals tend to become overly selective in identifying relevant information, ignore large portions of input, and struggle to connect detailed and overall perspectives. These patterns are characteristic of heuristic information processing. At the neural and behavioral levels, previous studies have shown that individuals with SCD differ in memory-related functional connectivity and working-memory efficiency, making them more susceptible to cumulative processing load and restricted selective attention in complex informational environments, which ultimately affects both attention and working memory (Viviano et al., 2019).

The prevalence of SCD is higher in women than in men, a finding also supported by the study of Xue et al. (2023). This difference may be because women are more sensitive to dynamic changes in cognitive function. Moreover, studies have shown that women are more likely to experience mental health issues such as depression, anxiety, and sleep disturbances, all of which are closely associated with SCD (Schliep et al., 2022). The onset of SCD–whether active or passive–may lead to reduced cognitive interference by filtering out irrelevant environmental stimuli, thereby enhancing attentional focus. However, this increased focus can also contribute to the accumulation of mental fatigue, ultimately leading to lower energy levels. SCD is also associated with slower information processing, which negatively impacts short-term memory efficiency and performance, resulting in poorer short-term memory compared to cognitively normal individuals (Yang et al., 2023). In addition, individuals with SCD tend to exhibit heightened self-awareness and monitoring of their cognitive performance. Due to diminished cognitive resources, individuals with SCD may find it difficult to fully assess all potential risks and rewards in decision-making contexts. This limitation may lead to neglect or misjudgment of risk-related information, increasing their tendency to make high-risk decisions. Furthermore, individuals with SCD are more likely to experience depressive symptoms, which are closely linked to lower self-efficacy and reduced problem-solving ability (Hill et al., 2021).

In addition, univariate analysis revealed that, compared to the healthy control (HC) group, individuals with SCD exhibited significantly higher levels of attention, Sleep Quality (SQ), Self-Perception (SP), Long-Term Memory (LTM), Depression Emotion (DE), and Psychological Self-Assessment (PSA), as well as significantly lower levels of Short-Term memory (STM) and Information Overload (IO) (all *P* < 0.05). These findings align with the seven key features identified through LASSO regression–Sex, Education Level (EL), STM, SP, Risk Decision (RD), DE, and IO, which are also recognized as important predictors of SCD. Given these associations, early assessment of psychological and cognitive factors, particularly Sex, EL, STM, SP, RD, DE, and IO, is recommended for individuals exhibiting SCD symptoms. Timely, targeted interventions may help improve the quality of life for patients with Parkinson’s disease. However, conventional screening tools are often limited by low efficiency, lack of standardization, and inconsistency across evaluations, making them time-consuming and labor-intensive. In contrast, intelligent assessment approaches offer a more efficient, accurate, and personalized means of evaluating cognitive function, thereby facilitating early detection, dynamic monitoring, and the development of tailored intervention strategies for individuals at risk of SCD.

Predictive models for SCD were developed using five machine learning algorithms: LR, Naive Bayes, SVM, decision tree, and neural network. The predictive performance of these models was systematically compared. SVM, a widely adopted supervised learning algorithm, is commonly used for classification, regression, and anomaly detection. It is particularly effective for high-dimensional data, as it constructs an optimal decision boundary by maximizing the margin, thereby minimizing the impact of dimensionality on model training and reducing the risk of overfitting. The findings of this study demonstrated that the SVM model outperformed the other algorithms, achieving an AUC of 0.90 in the training set and 0.82 in the internal validation set, with corresponding accuracies of 0.90 and 0.83. These results suggest that the SVM model provides superior discriminatory power and more accurate prediction of SCD risk.

In recent years, numerous studies have integrated neuroimaging with deep learning to improve the accuracy of early SCD identification. For example, Wang et al. (2020) extracted neuroimaging features using convolutional neural network (CNN), and Viviano et al. (2019) employed functional connectivity patterns to predict SCD risk. However, these studies often rely on high-cost data such as MRI or PET and require relatively large sample sizes. In contrast, the present study utilized psychological characteristics obtained from questionnaires and applied five machine learning algorithms, systematically evaluating their performance to select the optimal SVM model, which enhanced prediction accuracy while reducing overfitting and underfitting. Through LASSO regression and SHAP value analysis, the key information overload features contributing most to the prediction were identified, providing specific quantitative indicators that facilitate clinical assessment. This approach is well-suited for the early diagnosis and monitoring of SCD, offering high practicality and flexibility with lower cost. Nonetheless, the relatively small sample size may have introduced variability in model evaluation and selection, potentially compromising the accuracy of the chosen model and its parameters, and thereby affecting overall predictive performance. Future research should incorporate larger sample sizes to validate and extend the current findings. The SP scores in this study were derived from self-reports, which may be influenced by social desirability, emotional state, and individual differences in self-awareness, potentially leading to measurement bias. Future studies could include observer ratings and objective assessments, as well as conduct sensitivity analyses or longitudinal follow-ups. Moreover, all data were obtained from a single source, raising concerns about potential sampling bias. To enhance generalizability, future studies should include more diverse populations across geographic regions, age groups, genders, and socioeconomic backgrounds. Expanding the range of features–such as psychological traits, speech data, and neuroimaging biomarkers–may further improve the model’s robustness and applicability to real-world clinical settings.

## 5 Conclusion

This study successfully developed an SVM-based model for screening the risk of SCD. The SVM model demonstrated superior predictive performance compared to Naïve Bayes, Decision Tree, Neural Network, and traditional LR models. By facilitating the early identification of individuals at risk for SCD, the proposed model offers valuable support for clinical decision-making. Furthermore, it provides a novel framework for advancing the understanding and management of cognitive decline, with significant theoretical and practical implications.

## Data Availability

The raw data supporting the conclusions of this article will be made available by the authors, without undue reservation.

## Footnotes

1
https://zdjk.sanhaoyun.cn/
